# Clopidogrel-Induced Insulin Autoimmune Syndrome: Efficacy of Glucocorticoid Therapy and Continuous Glucose Monitoring

**DOI:** 10.2174/0118715303394353250727192242

**Published:** 2025-08-13

**Authors:** Lihui Luo, Xiaoqing Xiong, Jianmin Ran

**Affiliations:** 1 Department of Endocrinology and Metabolism, Guangzhou Red Cross Hospital of Jinan University, No. 396, Tong Fu Zhong Rd, Guangzhou 510220, China

**Keywords:** Insulin autoimmune syndrome, clopidogrel, hypoglycemia, drug adverse effect, glucocorticoid, continuous glucose monitoring

## Abstract

**Introduction:**

Insulin autoimmune syndrome (IAS) is a rare cause of spontaneous hyperinsulinemic hypoglycemia characterized by elevated insulin autoantibody (IAA). Over half of IAS cases involve exposure to sulfhydryl group-containing medications or their active metabolites, with clopidogrel being an uncommon trigger. We report a case of clopidogrel-induced IAS (CIAS) treated with glucocorticoid and managed by continuous glucose monitoring (CGM) during follow-up.

**Case Report:**

A 77-year-old man developed recurrent severe hypoglycemia events after receiving clopidogrel for six months. Laboratory investigations showed significantly elevated serum insulin levels (peak: 1,452.52 mIU/L; normal range: 1.9-23.0 mIU/L) and a high IAA titer of 37.0 COI (>1.1 COI, positive). Following the exclusion of other potential causes of hypoglycemia, IAS was diagnosed. Consequently, clopidogrel was discontinued, and combination therapy incorporating prednisolone was initiated. During the 10-month follow-up, insulin levels declined to 8.09 mIU/L with IAA titers decreasing to 1.35 COI. Analysis of CGM data demonstrated a transition from glycemic fluctuations to stabilization.

**Conclusion:**

This single-patient case report highlights the significance of identifying CIAS as a rare adverse effect of a drug, the effectiveness of glucocorticoid therapy, and the role of CGM in IAS management.

## 1 INTRODUCTION

Insulin autoimmune syndrome (IAS) is a rare autoimmune disease characterized by recurrent hypoglycemia resulting from raised insulin autoantibody (IAA) titers [1]. Although IAS has a low global incidence, over 85% of reported cases occur in East Asia, yet population-based prevalence remains undetermined due to a lack of epidemiological data [2]. The pathogenic mechanisms underlying IAS are multifaceted, involving complex interactions among genetic susceptibility, immunologic factors, and the use of sulfhydryl-containing drugs or exogenous insulin. Clopidogrel, a widely used antiplatelet aggregation agent, is metabolized by cytochrome P450 enzymes to generate immunogenic compounds containing sulfhydryl groups that may precipitate hypoglycemia [3, 4]. It has been reported that most patients experience symptom resolution within three to six months after stopping the culprit drugs [5]. Despite the increasing incidence of IAS, only 13 cases of clopidogrel-induced IAS (CIAS) have been reported in the literature to date, according to PubMed and Embase searches [3-14]. To highlight the diagnostic and therapeutic challenges of CIAS, we present an elderly male who developed refractory hypoglycemia despite combined therapy, including clopidogrel withdrawal, dietary management, and alpha-glucosidase inhibitors. Eventually, glucocorticoid therapy was initiated. However, there is no standardized treatment for CIAS, and clinicians tailor treatments based on the patient’s condition. This article aims to enhance awareness of CIAS by detailing the clinical presentation, therapeutic approach, and follow-up outcomes of a geriatric patient in our department.

## 2 CASE REPORT

In January 2024, a 77-year-old man with a BMI of 24.19 kg/m^2^ was admitted with recurrent hypoglycemia attacks for three months. Before admission, he experienced dizziness, palpitations, and fatigue occurring during both fasting and delayed postprandial states. His insulin release test demonstrated high insulin concentrations (Table **1**), with no history of exogenous insulin use. His medical history included hypertension and coronary atherosclerotic heart disease, for which he had been taking clopidogrel and atorvastatin for the past six months. He had moderate renal impairment but no liver disease or familial diabetes. Human growth hormone (GH), thyroid function, and cortisol rhythm were all within the normal ranges (Table **2**). Contrast-enhanced abdominal computed tomography (CT) excluded pancreatic tumors (Fig. **1**), and comprehensive serological testing showed no evidence of other autoimmune disorders. During the first three days of continuous glucose monitoring (CGM) data recorded by the SIBIONICS system, blood glucose levels fluctuated between 2.2 mmol/L and 15.2 mmol/L (normal range: 3.9-7.8 mmol/L), with hypoglycemia predominantly occurring at midnight and in the late postprandial phase. In one hypoglycemic episode, blood glucose was 2.4 mmol/L, insulin level was 995.07 mIU/L (normal range: 1.9-23.0 mIU/L), and C-peptide level was 6.97 nmol/L (normal range: 0.37-1.43 nmol/L). Critically, to further differentiate the diagnosis, an elevated IAA titer of 36.7 COI (>1.1 COI, positive) was detected, whereas no genetic variations were found through whole-exome sequencing. However, human leukocyte antigen (HLA) genotyping testing was not performed for personal reasons. These findings confirmed the diagnosis of IAS.

Initially, treatment consisting of small, frequent meals and acarbose therapy failed to prevent hypoglycemia. Clopidogrel, containing metabolized sulfhydryl groups, was suspected as the causative agent for IAS. Consequently, the drug was ceased, and prednisolone was initiated at 30 mg/day, tapered by 5 mg per week, and maintained at 5 mg/day until discontinuation in July (Fig. **2**). Upon follow-up after prednisolone withdrawal, his latest tests revealed a fasting insulin level of 8.09 mIU/L, a glycated hemoglobin (HbA1c) level of 6.2% (normal range: 0.0-6.1%), and a decrease in IAA titer from 36.7 COI to 1.35 COI in November. Until now, he has been treated with metformin to control blood glucose.

## 3 DISCUSSION

Currently, there are no recognized guidelines for the diagnosis of IAS, but the following criteria primarily informed it: first, patients frequently present with a typical Whipple’s triad; second, notwithstanding elevations in serum insulin and C-peptide levels, blood glucose levels during hypoglycemia attacks below 2.8 mmol/L; finally, the gold standard is a positive IAA test. Exclusion of all other plausible etiologies of hyperinsulinemic hypoglycemia is mandatory, encompassing insulinoma, non-insulinoma pancreatogenous hypoglycemia syndrome, and type B insulin resistance syndrome [7]. The present patient fulfilled the above criteria.

The incidence of IAS has progressively increased since its first report in 1970 [15]. So far, it is widely accepted that the production of IAA, characterized by high binding capacity and low affinity, is a central factor in IAS [1]. Mechanistically, upon binding with insulin, these antibodies rapidly form large antigen-antibody immune complexes after meals, thereby inducing postprandial hyperglycemia that accounts for the patient’s raised HbA1c level. In response, islet β-cells increase insulin secretion, while substantial amounts of free insulin are released into the blood due to dissociation of insulin-antibody complexes during the late postprandial phase, ultimately causing hypoglycemia. Since the association and dissociation of insulin from immune complexes are independent of blood glucose levels, the patient described here exhibited recurrent hypoglycemia and hyperglycemia. Moreover, hypoglycemic episodes occurred between 2 a.m. and 5 a.m., suggesting that insulin antibodies are sensitive to circadian variations in the internal environment.

Epidemiologically, a recent review documented 795 IAS cases worldwide, with the highest prevalence in Japan and China, respectively [2]. This geographic distribution correlates with the strong association between IAS and HLA-DR4, particularly DRB1*0406, an allele highly prevalent in Japan [16]. Although the absence of HLA genotyping in this case did not affect the diagnostic confirmation of IAS, it may have limited the complete immunogenetic characterization. Additionally, several sulfhydryl-containing drugs can cause IAS, possibly because of the sulfhydryl group’s ability to disrupt insulin disulfide bonds, among which clopidogrel represents a causative agent [3]. Of particular clinical relevance, a case review indicated that the average age at CIAS onset was 74.8 ± 8.6 years, with symptom onset ranging from one week to nearly two years following clopidogrel treatment [4]. Notably, the features observed in our patient align closely with the findings mentioned above (Table **3**).

The treatment schemes for CIAS patients included the withdrawal of causative drugs, dietary modifications, glucocorticoids [5-10], plasmapheresis [8], rituximab [17], and other interventions. While existing literature suggests that glucocorticoids do not significantly affect recovery time in CIAS, ticagrelor may be an alternative, although it is not routinely recommended [4]. Considering the patient's advanced age and preexisting cardiovascular/renal comorbidities, a moderate-dose glucocorticoid regimen (0.5 mg/kg/day prednisone equivalent) was initiated, consistent with published CIAS cases demonstrating effective autoimmune antibody suppression and a favorable safety profile [7, 9]. The glucocorticoid tapering protocol was individualized based on glycemic stabilization, reduction in both serum insulin levels and IAA titers from peak values, and absence of hypoglycemic episodes. This approach effectively balanced immunosuppression against risks of disease recurrence and steroid-related complications (notably gastrointestinal bleeding, adrenocortical insufficiency, and osteoporosis). Given the patient’s high bleeding risk (age > 65 years, hypertension, and renal dysfunction) and limited evidence for alternative antiplatelet agents in IAS, no replacement therapy was administered after clopidogrel discontinuation.

Glycemic management is a considerable challenge for IAS patients treated with glucocorticoids, as these agents can disrupt glucose metabolism. In this context, real-time CGM provides substantial benefits for monitoring and optimizing glucose control. Prior to prednisolone initiation, CGM detected alternating phenomena of hypoglycemia during fasting and delayed postprandial states, along with hyperglycemia in the early postprandial phase, accompanied by elevated glycemic variability (coefficient of variation, CV=37.27%, above the recommended 36% threshold) (Fig. **3A**). Guided by CGM data, we implemented a personalized nutrition intervention consisting of reduced meal portions during daytime and complex carbohydrate intake before bedtime, with dietary adjustments initiated when the time in range (TIR, glucose level: 3.9-7.8 mmol/L) fell below 70% or the time below range (TBR, glucose level <3.9 mmol/L) exceeded 3%. After prednisolone discontinuation, a 10-day CGM report showed improved TIR (89.6%), reduced TBR (0.8%), and lower CV (19.94%) (Fig. **3B**), suggesting a near absence of hypoglycemic attacks. Especially the observed decline in CV, which correlates with lower cardiovascular risk independent of HbA1c levels, underscores CGM’s unique capacity to simultaneously assess acute hypoglycemia risk through TBR monitoring, making it an essential tool for glycemic management in patients with significant glucose fluctuations [18]. In summary, CGM can develop individualized therapies based on real-time glucose data and promptly identify hypoglycemia, thereby reducing cardiovascular risks.

## CONCLUSION

In conclusion, our case report highlights the importance of recognizing IAS as a rare adverse effect associated with clopidogrel therapy. Particularly in geriatric patients exhibiting recurrent hypoglycemia, the differentiation of IAS from other etiologies poses a diagnostic challenge. The observed decline in serum insulin levels and IAA titers, coupled with the absence of further hypoglycemic episodes during follow-up, supports the efficacy of glucocorticoid therapy in alleviating IAS symptoms. For clinicians, prompt recognition of CIAS, along with immediate drug discontinuation, individualized consideration of glucocorticoids, and intensive glucose monitoring, is critical to accelerate clinical remission. Future multicenter studies are needed to establish optimal treatment protocols and explore safe antiplatelet alternatives to reduce the risk in IAS patients.

## Figures and Tables

**Fig. (1):**
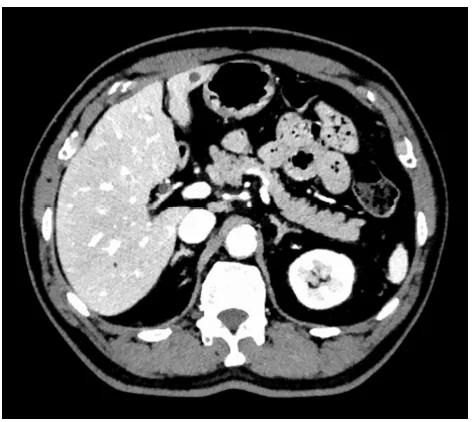
Contrast-enhanced abdominal computed tomography (CT) revealed no pancreatic tumors.

**Fig. (2):**
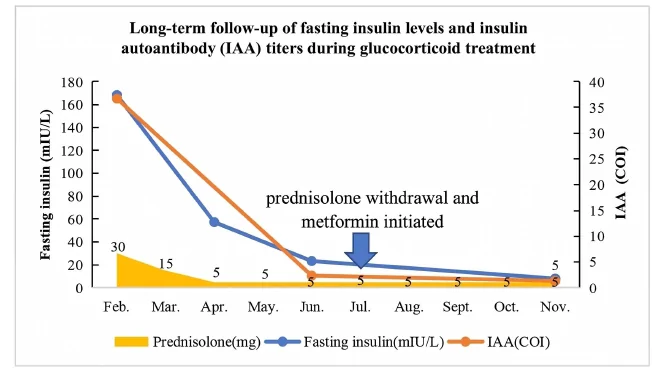
Fasting insulin levels (left Y-axis) and IAA titers (right Y-axis) showed a significant decline following prednisolone initiation (X-axis: time). Prednisolone was initiated at 30 mg/day, tapered by 5 mg weekly, and maintained at 5 mg/day until discontinuation in July.

**Fig. (3):**
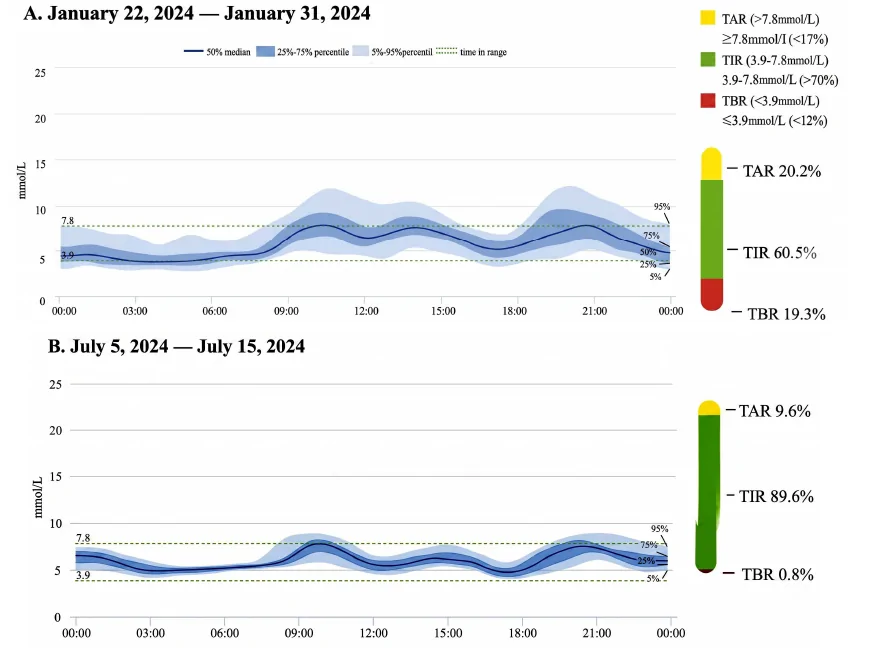
Comparison of ambulatory glucose profile (AGP) before and after glucocorticoid therapy. AGP analysis, generated through multi-day glucose data superposition and analyzed by specialized software, revealed distinct glycemic patterns: **(A)** Initial assessment demonstrated marked glucose variability with alternating hypoglycemia and hyperglycemia (TIR <70%, TBR >3%), indicating a significant hypoglycemia risk; **(B)** Post-treatment follow-up showed stabilized glucose control with normalized TIR and substantially reduced TBR (<1%), reflecting effective resolution of hypoglycemia episodes. The X-axis represents time, and the Y-axis represents blood glucose levels. TIR, time in range (glucose level: 3.9-7.8 mmol/L); TBR, time below range (glucose level <3.9 mmol/L); TAR, time above range (glucose level >7.8 mmol/L).

**Table 1:** Oral glucose tolerance tests (OGTT) and a 100-g standard steamed bread meal test showed elevated insulin levels.

**Insulin Release Test**	**Glucose (mmol/L)**	**Insulin (mIU/L)**
**OGTT (January 22^nd^, 2024)**		
0 h	5.6	168.34
2 h	18.6	1,452.52
**100-g standard steamed bread meal test (November 21^st^, 2024)**		
0 h	5.4	8.09
2 h	12.5	86.64

**Note:** OGTT, 75-g oral glucose tolerance test; Reference range: glucose: 0 h (3.9-6.1 mmol/L), 2 h (<7.8 mmol/L); insulin: 1.9-23.0 mIU/L.

**Table 2:** Laboratory examination at first admission (February 2^nd^, 2024).

**Items**	**Result**	**Reference Range**
HbA1c (%)	7.1 ↑	0.0-6.1
Cr (μmol/L)	126.0 ↑	62.0-115.0
eGFR (mL/min/1.73m^2^)	47.4↓	>90
ANA	Negative	Negative
FT3 (pmol/L)	4.32	3.10-6.80
FT4 (pmol/L)	15.6	12.0-22.0
TSH (mIU/L)	2.26	0.27-4.20
Cortisol (nmol/L)	503.84 (8:00 am)/254.67 (4:00 pm)/86.23 (12:00 am)	Am 185-624, Pm<276
ACTH (pmol/L)	9.8(8:00 am)/6.55 (4:00 pm)/2.46 (12:00 am)	1.6-13.9
24h urine phosphorus (nmol/24h)	747.21	160-1088
GH (ng/mL)	0.26	Male, 20-79 years old, 0.03-2.47
IGF-1(ng/mL)	155.00	35-216
IAA (COI)	36.7 ↑	>1.1, positive
ICA (COI)	0.05	<0.9
GADA (IU/ml)	0.98	<10
IA-2A (IU/ml)	<0.70	<10

**Abbreviations:** HbA1c, glycated hemoglobin; Cr, creatinine; eGFR, estimated glomerular filtration rate; ANA, antinuclear antibody; FT3, free triiodothyronine; FT4, free thyroxine; TSH, thyroid-stimulating hormone; ACTH, adrenocorticotrophic hormone; GH, growth hormone; IGF-1, insulin-like growth factor-1; IAA, insulin autoantibody; ICA, islet cell antibody; GADA, glutamic acid decarboxylase antibody; IA-2A, insulinoma-associated antigen 2 antibody.

**Table 3:** Comparative analysis of the present case and published CIAS cases.

**Differentiation Point**	**Published CIAS Cases (n=13)**	**Present Patient**
High-risk population	East Asian (n=8), especially Japanese(n=3) and Chinese (n=5)	Chinese
Sex	Male (n=11), Female (n=2)	Male
Age (years)	72.1 ± 9.3	77
Symptom onset	1 week - 2 years	6 months
Laboratory	**Elevated Insulin, Positive IAA**	
Therapy	● Clopidogrel discontinuation ● Small frequent meals ● ɑ-Glucosidase inhibitor ● Glucocorticoids ● Plasma exchange ● Rituximab treatment	Clopidogrel discontinuation, small frequent meals, and prednisolone treatment.
Response to therapy	**All Cases Achieved Remission After Drug Discontinuation ± Other Therapies**	
Use of CGM	4/13 cases	Yes
Alternative antiplatelet drug	Aspirin (n=1), ticagrelor (n=2), prasugrel (n=1)	None

**Note:** The data on age are shown as mean ± SD; **Abbreviations:** CIAS, clopidogrel-induced insulin autoimmune syndrome; IAA, insulin autoantibody; CGM, continuous glucose monitoring.

## LIST OF ABBREVIATIONS

IAS: Insulin Autoimmune Syndrome
IAA: Insulin Autoantibody
CIAS: Clopidogrel-induced Insulin Autoimmune Syndrome
CGM: Continuous Glucose Monitoring
BMI: Body Mass Index
GH: Growth Hormone
CT: Computed Tomography
HLA: Human Leukocyte Antigen
HbA1c: Glycated Hemoglobin
TIR: Time In Range
TBR: Time Below Range
CV: Coefficient of Variation
TAR: Time Above Range

## STANDARDS OF REPORTING

CARE guidelines were followed.
